# Clinical application of individualized 3D-printed navigation template to children with cubitus varus deformity

**DOI:** 10.1186/s13018-020-01615-8

**Published:** 2020-03-19

**Authors:** Xinyue Hu, Meiling Zhong, Yue Lou, Peng Xu, Bo Jiang, Fengyong Mao, Dan Chen, Pengfei Zheng

**Affiliations:** Department of Orthopaedic Surgery, Children’s Hospital of Nanjing Medical University, Nanjing, 210000 Jiangsu Province China

**Keywords:** Individualized navigation template, 3D-printing technology, Cubitus varus deformity, Children

## Abstract

**Background:**

Cubitus varus deformity is a common sequela of elbow fractures in children. Cubitus varus deformity treatment is tending toward 3D correction, which is challenging for orthopedic surgeons. This study aims to explore whether individualized 3D-printed navigation templates can assist with accurate and effective corrective treatment of children with cubitus varus deformity.

**Methods:**

Thirty-five patients were treated for cubitus varus deformity from June 2015 to April 2017, including 21 boys and 14 girls, aged 4.6–13.2 years (average, 7.5 years). Of these cases, 17 deformities were on the left side and 18 were on the right side. All were treated with wedge osteotomy of the lateral distal humerus. 3D-printed navigation templates were used in 16 cases, while traditional surgery was used in 19 cases. All patients underwent computed tomography scans before surgery. Computer software was used to analyze the measurements and design and print individualized navigation templates. The navigation templates were matched, and surgery was initially simulated. Intraoperative individualized navigation templates were used to assist with accurate osteotomy and Kirschner wire fixation. Operation times were recorded in all cases, the carrying angles before and after surgery were assessed by computer, and postoperative elbow joint function was evaluated using Bellemore criteria. All measurement data were presented as means ± SD, and Student’s *t* test was used to examine differences between groups. All count data between both groups were compared using the chi-square test or Fisher’s exact test analysis.

**Results:**

All individualized navigation templates matched well with the corresponding anatomical markers and were consistent with preoperative planning, simulated surgery, and intraoperative procedures. Average operation times from clear exposure to fixed Kirschner wire were 11.69 min (9.6–13.5 min) for the individualized navigation template group and 22.89 min (17.7–26.8 min) for the traditional operation group (*p* < 0.001). Average differences in postoperation carrying angles between affected and healthy sides were 1.13° (0–2.0°) and 4.21° (0–7.5°), respectively (*p* < 0.001). Follow-up 6–12 months postoperation showed that elbow function did not differ significantly between groups using the Bellemore criteria (*p* > 0.05).

**Conclusions:**

Individualized navigation templates simplify procedures, reduce operation time, and improve accuracy when used in orthopedic surgery to treat children with cubitus varus deformity.

## Background

Cubitus varus deformity is a common sequela of elbow fractures in children [1–4]. Although the cause of cubitus varus deformity remains inconclusive, it is considered a three-dimensional (3D) deformity including varus deformity of the coronal plane, an overextension deformity of the sagittal plane, and an internal rotation deformity of the horizontal plane [5, 6].

Until recently, most orthopedic surgeons only performed humeral supracondylar valgus osteotomy to treat cubitus varus deformity [1, 7], believing that the rotational deformity of the horizontal plane did not need to be corrected owing to shoulder joint compensation [8, 9]. However, some researchers think that the biomechanical axis disruption includes internal rotation deformity, which leads to soft tissue and morphologic bony alterations in the elbow and offers a compelling argument for corrective osteotomy to treat pediatric cubitus varus [10–13].

Lateral wedge osteotomies are performed most commonly [2]. This technique is more simple, effective, and reproducible than other osteotomy styles, such as dome osteotomy and multiplanar osteotomy. The main drawback of lateral wedge osteotomy is the prominence of the lateral condyle and the asymmetry of the two osteotomy surfaces, which might result in a step at the end of the osteotomy.

Treatment for cubitus varus deformity is now trending toward 3D correction [14], but this technique is challenging for orthopedic surgeons [15–18]. Accurately controlling the correction angle of each dimension during the operation is difficult, and the degree of correction must often be repeatedly adjusted during surgery or determined based on general appearance. Therefore, errors often occur in the preoperative planning and postoperative effect, resulting in an unsatisfactory surgical effect.

In recent years, the application of 3D-printed technology in the medical field has developed rapidly, especially that of individualized navigation templates, which are widely used in many clinical disciplines [19–23]. Our previous research has applied 3D-printed individualized navigation templates to the treatment of children with orthopedic diseases, such as femoral neck fractures and developmental dysplasia of the hip [24]. Therefore, we conducted a prospective randomized study of children with cubitus varus deformity admitted to our hospital from June 2015 to April 2017, who were randomly divided into a traditional surgery group and a 3D individualized navigation template group. This study aimed to explore whether individualized 3D-printed navigation templates can assist with accurate and effective corrective treatment of children with cubitus varus deformity.

## Methods

### General information

Thirty-five cases of cubitus varus deformity were included in the study. The patients were 21 boys and 14 girls, aged 4.6–13.2 years (average, 7.5 years). The deformity was present on the left in 17 cases and on the right in 18 cases. The injuries were caused by supracondylar fracture of the humerus. After fracture, 24 cases underwent closed reduction and percutaneous Kirschner wire fixation, and 11 underwent manual reduction of plaster fixation. Patients that fulfilled the following criteria were eligible for the study: (i) all elbow varus deformities were secondary to the supracondylar fracture, (ii) all affected sides had carrying angles of more than − 15°, and (iii) the timing of cubitus varus orthopedic surgery was 12–43 months after the fracture occurred (mean, 16.4 months). Patients were excluded from the study when (i) the patient suffered from metabolic bone disease, (ii) other fractures were present, and (iii) the patient had previously undergone corrective surgery due to cubitus varus deformity. All patients were treated with wedge osteotomy of the lateral distal humerus. In 16 cases (8 on each side), 3D-printed navigation templates were used; in 19 cases (9 on the left and 10 on the right), traditional surgery was performed. Computed tomography (CT) scanning data of the bilateral upper extremities were collected from patient records. Preoperative CT images were acquired on a 64-MSCT scanner (Philips, Netherlands) with parameters of 120 kV, 160 mA, and 1-mm section thickness. Imaging data were stored in DICOM format. The hospital ethics committee approved this study, and the legal guardians of the children gave informed consent.

### Design and printing of individualized navigation templates

**Fig. 1:**
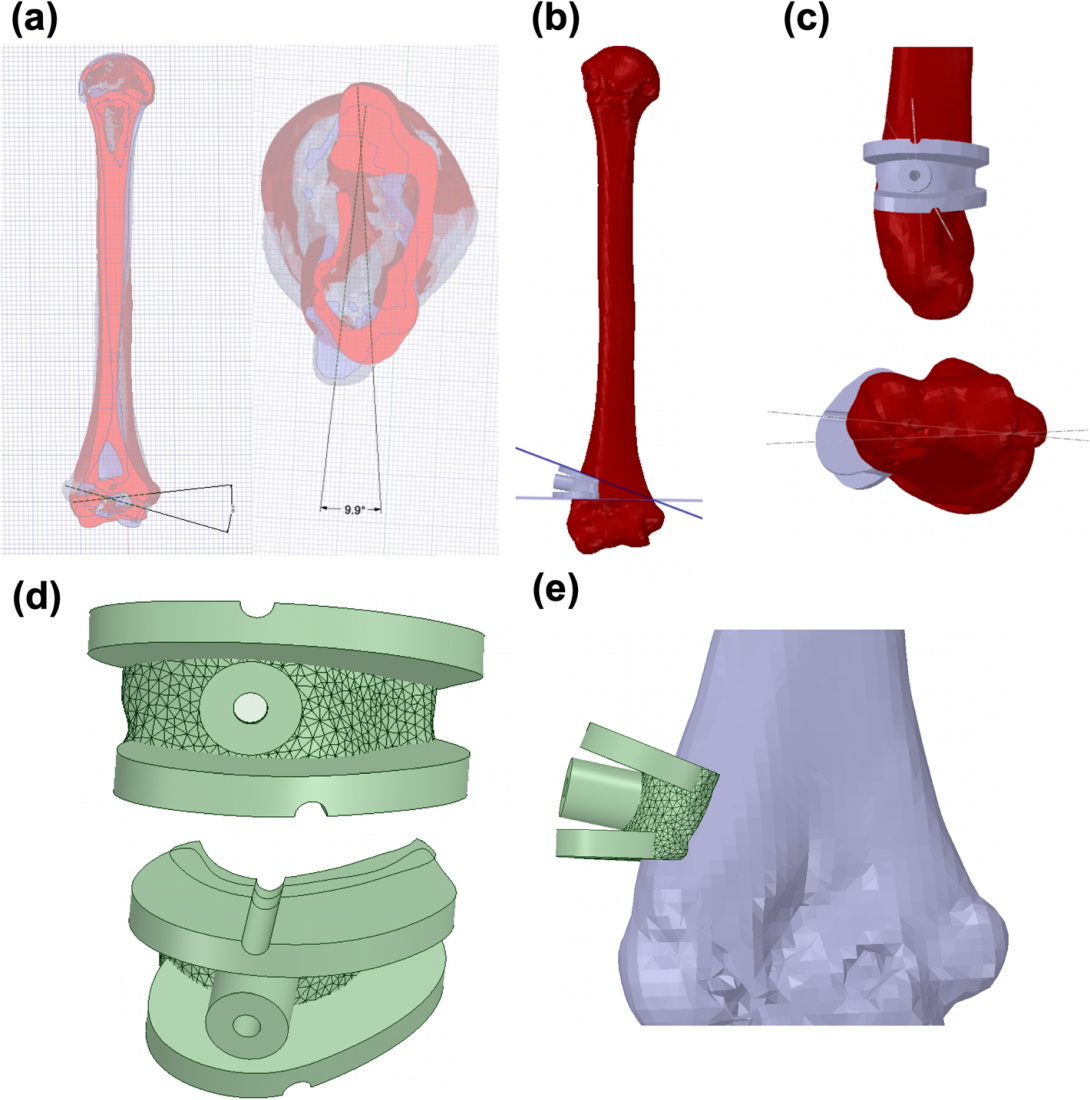
Preoperative computer simulation of the model and design of the navigation template. **a** Bilateral elbow joint parameters were accurately measured via 3D reconstruction of the CT scanning data. **b** Angles to be turned out and rotated were determined. **c** A 5-mm-thick process in the opposite direction was used to create a matching substrate, while the Kirschner wire guide pipe data were imported, combined, and reconstructed into an individualized navigation template. The rotation angle was also calculated, and the positions of the upper and lower positioning pipes of the guide plate were determined. **d**, **e** After passing the Boolean operation, the guide plate design was completed

CT scanning data were imported into a computer, and the 3D reconstructed upper limb models were generated using Mimics 17.0 software (Materialise NV, Leuven, Belgium). The angle of the coronal osteotomy was calculated by computer software from the image overlap of the affected side and the contralateral side, and then, the angular difference between the two at the distal end of the humerus was directly measured. This angle difference was the angle at which the osteotomy was required (Fig. 1a). The two osteotomy planes were designed as isosceles triangles to ensure that the lateral edge of the osteotomy plane was continuous and did not form a step [25]. The lateral osteotomy plane was approximately 1.0 cm above the olecranon fossa (Fig. 1b). A 5-mm-thick process in the opposite direction was used to create a matching substrate, while the Kirschner wire guide pipe data were imported, combined, and reconstructed into an individualized navigation template. The rotation angle was calculated, and the positions of the upper and lower positioning pipes of the guide plate were determined (Fig. 1c). After completing the osteotomy, the two pipes were rotated and coincided to correct the distal internal rotation deformity. After passing the Boolean operation, the Kirschner wire guide pipe was penetrated to complete the guide plate design (Fig. 1d), the data were imported into the 3D printer, and the distal humerus model and navigation template (Fig. 1e) were printed using the medical polylactic acid (PLA) material.

### Preoperative testing of the cubitus varus deformity model and individualized navigation template

For the distal humerus wedge osteotomy, after completely matching the individualized navigation template with the distal end of the humerus, the navigation template was fixed with a Kirschner wire to prevent slippage. The upper and lower positioning pipes of the osteotomy surface in the navigation template were marked with a Kirschner wire. A wedge-shaped osteotomy was then performed along the upper and lower osteotomy surfaces of the navigation template. The wedge-shaped osteotomy block was removed along with the fixed Kirschner wire and navigation template. The distal and proximal ends of the osteotomy surface were then joined, and the distal end was rotated outward to coincide with the two marked holes. Finally, two Kirschner wires were crossed to fix the distal and proximal ends of the osteotomy.

### Operation and postoperative treatment

**Fig. 2:**
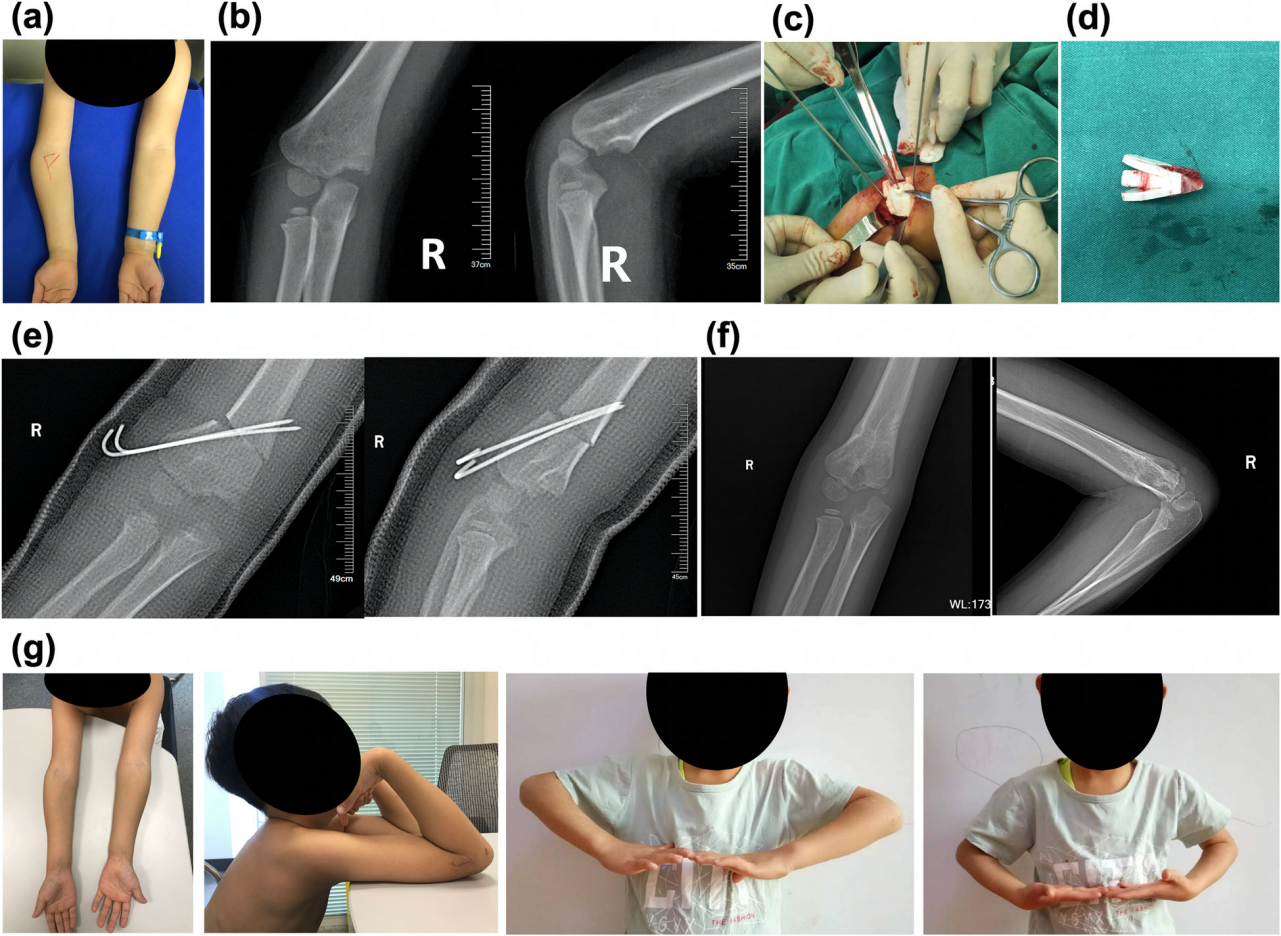
Radiographs and comparison between preoperative and intraoperative appearance. **a** Appearance showing different carrying angle of bilateral upper limbs and varus deformity of the right elbow. **b** Posteroanterior radiograph of right elbow joint showing cubitus varus deformity before operation. **c** Individual osteotomy navigation template matched the distal humerus well, and fixation of the navigate template with one Kirschner wire; two holes were drilled with the Kirschner wire to control rotational rectification. **d** Osteotomy was performed according to the navigation template surface, and the osteotomy block was completely consistent with the preoperative design. **e** Lateral radiograph of right elbow joint showing no extension or flexion deformity after operation. **f** Postoperative appearance showing correction of the deformity 10 weeks after operation. **g** Appearance showing correction of the deformity after operation, with no limit to the flexion and extension of the elbow joint and rotation of the forearm

Patients (Fig. 2a, b) were anesthetized and placed in a supine position, and the affected elbow was exposed in vitro. The skin, fascia, and muscle layers were cut; then, the periosteum was dissected to expose the lateral cortical bone of the distal humerus. After matching the navigation template to the distal end of the humerus, the navigation template was fixed with a Kirschner wire to prevent slippage. The upper and lower positioning pipes of the osteotomy surface of the navigation template were marked with a Kirschner wire (Fig. 2c). A wedge-shaped osteotomy was performed along the upper and lower osteotomy surfaces of the navigation template. The wedge-shaped osteotomy block, the Kirschner wire for fixation, and the navigation template were then removed together (Fig. 2d). For the distal and proximal ends of the osteotomy plane, the distal end was rotated outward until the two marked holes coincided. The distal and proximal ends of the osteotomy were cross-fixed with two Kirschner wires, and the affected limb was observed. When the limb appearance was satisfactory, the surgical field was washed and sutured in layers. The elbow joint was placed at 20° flexion, and the long arm plaster was fixed to complete the operation. X-rays were reviewed regularly after surgery (Fig. 2e, f). At 6 weeks postoperation, continuous bone callus shadows were seen at the osteotomy site, and the osteotomy line was blurred. Therefore, the plaster and Kirschner wire were removed. The patients were instructed to perform functional exercises on the elbow joint. The carrying angle and elbow joint mobility were measured at follow-up (Fig. 2g). The carrying angle of cubitus varus deformity was recorded as a negative value. The treatment effect was evaluated according to Bellemore criteria [26], which was divided it into three levels, as follows: excellent, difference between the affected side and normal side of < 5° with elbow joint flexion and extension motion limited to < 10°; good, difference between the affected side and normal side of 6–10° with elbow joint flexion and extension motion limited to 11–20°; and poor, remaining varus deformity with difference between the affected side and normal side of > 20°, or complications requiring surgery.

### Statistical analysis

All measurement data were presented as means ± SD, and Student’s *t* test was used to examine the differences between groups. All count data of the two groups were compared using the chi-square test or Fisher’s exact test analysis. All statistical analyses were conducted using Stata software version 9.0 (StataCorp LP, College Station, TX, USA) and SPSS software version 17.0 (SPSS Inc., USA). Statistical significance was set at *p* < 0.05.

## Results

### Preoperative computer simulation of the model, preparation of the navigation template, and simulated surgery results

The bilateral elbow joint parameters were accurately measured via 3D reconstruction of the CT scanning data (Fig. 1a). The angles that needed to be turned out and rotated were determined (Fig. 1b, c); then, a computer was used to simulate the osteotomy plane and the fixed needle of the Kirschner wire. Navigation templates were successfully designed for all patients. All navigation templates matched the distal humerus model exactly in simulated surgery. The osteotomy was successfully completed along the direction of the osteotomy surface of the navigation template, and the osteotomy block was removed. The two ends of the model were rotated after the osteotomy to coincide with the positioning points, then fixed with two crossed Kirschner wires. The appearance of the distal humerus returned to normal, which was consistent with the computer planning and design results.

### Surgical results and postoperative evaluation

For the 16 children with cubitus varus deformity that received the 3D-printed individualized navigation template, the intraoperative navigation template and the distal radius model were completely matched (Fig. 2c), and the osteotomy along the navigation template was successful. The time from initial placement of the navigation template to secure fixation of the two Kirschner wires was 11.69 ± 2.21 min. Patients were followed up with for 6–12 months after surgery (Table 1). The postoperative appearances of the patients were well corrected, and the prominence of the lateral condyle was significantly improved compared with preoperation (Fig. 2g). Anteroposterior and lateral radiographs showed no varus, extension, or flexion deformity 10 weeks after operation (Fig. 2f). The carrying angle of the affected side preoperation was − 23.50 ± 5.75°, while the corresponding healthy side carrying angle was 5.19 ± 2.79°. The carrying angle of the affected side postoperation was 4.94 ± 1.53°, and the difference in angle to that of the healthy side was 1.13 ± 1.20°. At the final follow-up, the maximum extension angle of elbow joint mobility on the affected side was 1.00 ± 6.24°, and the maximum flexion angle was 126.3 ± 5.33°. With respect to the Bellemore criteria, 14 cases were excellent and two cases were good. No complications, such as incision infection, nonunion, neurovascular injuries, pin tract infection, and loss of reduction, occurred.

**Table 1:** Comparison of general information, operation information, and results relating to the navigation template group and conventional surgery group. Operation time is from clear exposure to the fixed Kirschner wire

	Cases	Age	Gender		Side		Operation time (min)	Postoperative appearance		Carrying angle (degrees, °)				Maximum elbow motion (degrees, °)		Bellemore criteria [26]		
		(year, *X* ± *S*)	M	F	L	R		Satisfied	Not satisfied	Affected side before operation	Normal side	Affected side after operation	Difference in range between affected and normal elbow	Extension	Flexion	E	G	P
Navigation template group	16	6.86 ± 1.84	9	7	8	8	11.69 ± 2.21	16	0	− 23.50 ± 5.75	5.19 ± 2.79	4.94 ± 1.53	1.13 ± 1.20	1.00 ± 6.24	126.3 ± 5.33	14	2	0
Conventional surgery group	19	7.79 ± 2.51	12	7	9	10	22.89 ± 3.94	16	3	− 25.26 ± 5.35	5.21 ± 2.37	3.94 ± 3.25	4.21 ± 2.27	2.00 ± 6.51	126.8 ± 5.08	12	7	0
*p* value		0.224	0.678		0.877		< 0.001	0.234		0.355	0.979	0.272	< 0.001	0.648	0.789	0.101		

*M* male, *F* female, *L* left, *R* right, *E* excellent, *G* good, *P* poor)

In the 19 children with cubitus varus deformity that received traditional surgery, the time from clear exposure to completion of the Kirschner wire fixation was 22.89 ± 3.94 min. Although the varus deformity was well corrected by traditional surgery, the prominence of the lateral condyle became more apparent. The carrying angle of the affected side preoperation was − 25.26 ± 5.35°, while that of the healthy side was 5.21 ± 2.37°. The carrying angle of the affected side postoperation was 3.94 ± 3.25°, and the difference in angle to that of the healthy side was 4.21 ± 2.27°. At the final follow-up, the maximum extension angle of the elbow joint mobility on the affected side was 2.00 ± 6.51°, and the maximum flexion angle was 126.8 ± 5.08°. With respect to the Bellemore criteria, 12 cases were excellent, and seven cases were good. No complications, such as incision infection, nonunion, neurovascular injuries, pin tract infection, or loss of reduction, occurred in any of the 19 cases. The operating time for the patients who received the 3D-printed navigation template was significantly shorter than that of the patients who underwent traditional surgery (*p* < 0.001). In the 3D-printed navigation template group, the difference between the affected side and the healthy side was significantly smaller than that of the traditional surgery group (*p* < 0.001). Elbow joint function did not significantly differ between the two groups (*p* > 0.05).

## Discussion

Scholars increasingly believe that the cubitus varus deformity is a 3D deformity, including varus deformity of the coronal plane, overextension deformity of the sagittal plane, and internal rotation deformity of the horizontal plane [5]. This poses a challenge for orthopedic surgeons. Accurately controlling the correction angle in each dimension during the operation is difficult, and the adjustment must often be repeated during surgery or the degree of correction must be determined based on general appearance [27]. Medical 3D-printing technology is based on computer-aided design (CAD) and rapid prototyping technology [28, 29], using medically specific materials to print products that can be applied in the medical field and have individualized 3D structures or functions [30]. With increasing demand for individualized medical treatment, 3D-printing technology applications and research are developing rapidly [31]. Specifically, 3D-printed surgical navigation templates are widely used in orthopedics owing to simple operation and practicality [20–24]. Individualized navigation templates based on reverse engineering and 3D-printing technology can completely match the solid skeleton of a specific case and allow accurate control of the plane and angle of the osteotomy, which is safe and accurate [32]. As children have large individual differences, their orthopedic surgery requires the assistance of surgical navigation templates to improve surgical accuracy and results and reduce complications and sequelae.

In our study, the individualized navigation template designed via CAD was wedge-shaped, making it simple in design and convenient in surgery, and had a small volume that did not expand the surgical incision. The template included a fitting surface and upper and lower osteotomy surfaces. The angle between the upper and lower osteotomy surfaces was equal to the correction of the deformity angle. A Kirschner wire guide pipe was attached to the fitting surface to fix the navigation template and prevent errors due to intraoperative slippage. The Kirschner wire guide pipe on the osteotomy surface provided a mark for correcting the rotational deformity. The individualized 3D-printed surgical navigation templates showed a high degree of matching with the distal end of the humerus. The osteotomy was successfully performed under the guidance of the navigation template, which prevented repeated adjustments, making the operation more convenient. Evaluating postoperative elbow function using Bellemore criteria showed no statistical difference between the patients using the individualized navigation template and those who underwent traditional surgery. The operation time for the navigation template group was significantly reduced, and the difference in the carrying angle between the affected and healthy elbow joints was smaller both preoperatively and postoperatively. The navigation template had the advantages of speed, convenience, and accuracy. The insignificant difference in elbow function between the groups might be due to the insufficient number of cases and short follow-up time. Some researchers have tried to use individualized navigation templates to assist with cubitus varus osteotomy. Zhang et al. [33] obtained satisfactory results using a CAD navigation template on cubitus varus deformity, but this template only corrected the varus deformity. Omori et al. [16] designed a surgical navigation template to correct the 3D deformity, but its large size made the surgical incision larger than that of conventional surgery, which was not easily accepted by patients.

Summarizing the clinical treatment experience showed that this new navigation template-assisted treatment for children with cubitus varus deformity had the following advantages. First, it enabled individualized patient data to be measured by computer to determine the osteotomy angle, osteotomy plane, rotation angle, and other parameters to yield the best orthopedic effect. Second, it prevented damage of the surrounding tissues and epiphyseal cartilage, which reduced the complication incidence. Third, applying the individualized surgical navigation templates was simple and convenient, and the template could be closely attached to the corresponding anatomical structure to accurately position and orient the surgical area, which saved time during the operation. Furthermore, this template can shorten the learning curve for new clinicians and assist them in training for surgical operations. Finally, and most importantly, a computer-assisted method can be used to design an isosceles triangle navigation template with a perfectly symmetrical upper and lower osteotomy. After the osteotomy, the upper and lower osteotomy surfaces were completely fitted, which avoided the steps on the upper and lower osteotomy surfaces that often occur after osteotomy using the traditional surgical method. Furthermore, the 3DP navigation template used in this study also assisted the surgeon in correcting the internal rotation deformity by externally rotating the osteotomy end of the distal humerus according to the results of preoperative computer measurements and the markers on the 3DP template during surgery. This can rotate the prominence of the lateral condyle to the posterior elbow, such that biomechanical changes in the internal rotation deformity are corrected, and the convexity of the outer lateral ankle is significantly improved.

However, this method also has some disadvantages. First, the friction of the chainsaw tended to deform the osteotomy surface of the navigation template, which could affect operation accuracy. The process of passing the Kirschner wire through the guide pipe will produce wear on the pipe, and heat can damage the pipe structure, affecting the accuracy of rotation alignment and increasing the chance of surgical contamination. During the operation, the surgical field should be more heavily irrigated to avoid debris residue. Second, correcting the sagittal deformity of the sagittal plane was not reflected in the surgical navigation template because the direction of the sagittal deformity is consistent with the direction of the elbow joint. The patient has great self-shaping ability in the deformity consistent with the direction of joint movement [34, 35]. In the intraoperative incision, the angle between the two osteotomy sections can be controlled more clearly. In future research, we will focus on controlling these parameters. Finally, the new technique allows for simultaneous correction in the rotational and coronal plane, which might increase the risk of other complications, such as neurovascular injury and nonunion, which should be studied with a larger sample and longer follow-up.

This study also has some limitations: First, the number of cases that received the navigation template for the osteotomy operation was small, the follow-up time was insufficient, and the results were not compared with those of the traditional surgery group over the long-term. Future research will focus on a larger sample and long-term follow-up studies with application of the template. Second, this method was a closed wedge osteotomy, which might produce shortening of the limb. To avoid this, we might design another osteotomy method in the future. Third, we did not do a precise cost-effectiveness analysis which is important for a new technology. By using this technology, patients needed to pay about $150 for navigation template design and printing, but due to the reduction of operation time and the reduction of X-ray times during the operation, the operation cost was reduced about $160. However, more data (such as direct cost, indirect cost, invisible cost) are needed for cost-effectiveness analysis, especially long-term follow-up is needed to compare whether the patients in the two groups need corresponding drugs or surgical treatment for elbow joint pain in the later 30 or 40 years, which will cause more costs.

## Conclusions

In summary, a new individualized navigation template constructed using CAD and 3D-printing technology has been used to assist in orthopedic surgery for children with cubitus varus deformity. This template can simplify the surgical procedure, reduce the operating time, improve the surgical accuracy, and shorten the learning curve of new clinicians. Therefore, this method is worthy of popularization and application.

## Data Availability

The datasets used and/or analyzed during the current study are available from the corresponding author on reasonable request.

## Abbreviations

3D: Three-dimensional
CAD: Computer-aided design
CT: Computed tomography
PLA: Polylactic acid
